# Intestinal microbiota and their metabolic contribution to type 2 diabetes and obesity

**DOI:** 10.1007/s40200-021-00858-4

**Published:** 2021-08-04

**Authors:** A. L. Cunningham, J. W. Stephens, D. A. Harris

**Affiliations:** 1 Department of Surgery, Swansea Bay University Health Board, Singleton Hospital, Swansea, UK; 2 School of Medicine, Swansea University Medical School, Swansea University, Institute of Life Science 2, Swansea, UK

**Keywords:** Gut microbiota, Obesity, Type two diabetes mellitus

## Abstract

Obesity and type 2 diabetes mellitus (T2DM) are common, chronic metabolic disorders with associated significant long-term health problems at global epidemic levels. It is recognised that gut microbiota play a central role in maintaining host homeostasis and through technological advances in both animal and human models it is becoming clear that gut microbiota are heavily involved in key pathophysiological roles in the aetiology and progression of both conditions. This review will focus on current knowledge regarding microbiota interactions with short chain fatty acids, the host inflammatory response, signaling pathways, integrity of the intestinal barrier, the interaction of the gut-brain axis and the subsequent impact on the metabolic health of the host.

## Background

Obesity is an increasing global challenge with a current worldwide estimation of 1.9 billion adults being classed as overweight (body mass index (BMI) > 25), 650 million of which are obese (BMI > 30) with the total currently outnumbering those with malnutrition [1, 2]. Key factors contributing to the current obesity epidemic include:—1) an increased availability and intake of energy-dense foods that are high in fat and sugars; 2) a significant decrease in physical activity; and 3) the evolving interactions of host-intestinal microbiota and environment. Obesity is a significant risk factor for prevalent non-communicable conditions such as cardiovascular disease, diabetes, musculoskeletal disorders and several types of malignancy. The risk for these disorders increases exponentially with increasing BMI underlying the need for early recognition and intervention.

The number of people being diagnosed globally with type 2 diabetes mellitus (T2DM) is projected to reach beyond 700 million within the next twenty-five years [3]. Importantly, the prevention of T2DM has been identified and declared a target priority by the World Health Organization (WHO) [4] and the United Nations (UN) [5]. Increasing weight, central body fat distribution and BMI play an integral role in the development of T2DM, a chronic metabolic disease characterised by hyperglycaemia and associated with insulin resistance and/or insufficient pancreatic insulin production [6]. Obesity alone accounts for 80–85% of the risk factors for T2DM [7]. Similar to obesity, the complications of T2DM include cardiovascular disease, kidney disease, limb amputations and blindness, which all subsequently can lead to disability and premature mortality with a global healthcare cost of greater than 1.3 trillion dollars [8].

## Gut microbiota

Hippocrates, the ‘father of medicine’, claimed ‘all disease begins in the gut’ [9] indicating that the contribution to human health that the intestinal microbiota provides has been thought about for thousands of years. However, only recently, has the acknowledgement been stated that, as humans, we are in fact ‘supra-organisms’ composed of both human and microbial cells [10]. Humans carry two sets of genes, those encoded in our own genome and those encoded in our microbiota. Metchnikoff, in the early 1900s, first suggested the central importance of the intestine in host physiology and pathology [11]. Intestinal microbiota were viewed as essential modulators with the ability to influence human homeostasis and that disruption of this harmony by specific microbiota could result in a diseased state through accumulation of microbiota by-products.

The ‘human microbiome’ was first postulated by Joshua Lederberg, to signify the ecological community of commensal, symbiotic and pathogenic microorganisms that share our body space [12]. The genetic material of the intestinal microbes, collectively defined the ‘gut microbiome’, surpasses the magnitude of the human genome over one hundred times [13, 14]. Many bacterial species cannot be cultured, but with the recent technological advancement of modern molecular methods has led to the evolution of next-generation sequencing technology for the study of microbial deoxyribonucleic acid (DNA) from faecal samples. This has provided the ability to examine the entire genomic content of a community, by using direct sequencing of microbial RNA without the need for prior amplification [15–17].

An adult human is colonized by approximately one hundred trillion microbes, most of which are predominantly found in the gastrointestinal tract (GIT). Accommodating this enormous number of microorganisms in the GIT has often led to the intestinal microbiota being referred to as the ‘hidden organ’ [18]. The GIT hosts bacteria, archaea, viruses and fungi, with the largest population of microbiota being found to inhabit the colon. Gastric acid, bile and pancreatic secretions prevent the colonization of the stomach and proximal small intestine by most bacteria. Bacterial density begins to increase towards the distal small intestine (10^8^ bacteria per gram content in the distal ileum), and increases rapidly throughout the colon rising to an estimated 10^11^ – 10^12^ bacteria per gram of colonic content, contributing roughly 60% of faecal mass [15, 19] estimated to weigh approximately 1.5 kg (kg) [20].

**Fig. 1:**
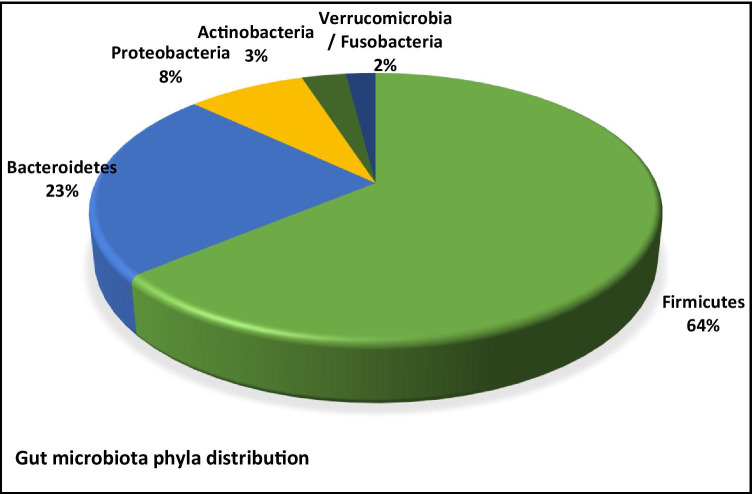
Colonic microbiota belong to the main phyla in the following order: *Firmicutes* (64%), *Bacteroidetes* (23%), *Proteobacteria* (8%), *Actinobacteria* (3%), *Verrucomicrobia* and *Fusobacteria* (2%)

Over 90% of all phylotypes of colonic bacteria belong to just two known phyla; Gram-positive *Firmicutes* and Gram-negative *Bacteroidetes*. Bacteria are estimated to belong to over five hundred different species with 99% belonging to just thirty to forty genera from the four main phyla: *Firmicutes* 64% (e.g. *Clostridium*, *Enterococcus*, *Lactobacillus*, *Ruminococcus*); *Bacteroidetes* 23% (e.g. *Bacteroides*, *Prevotella*); *Proteobacteria* 8% (e.g. *Helicobacter*, *Escherichia*); and *Actinobacteria* 3% (e.g. *Bifidobacterium*) [21–23]. Other, much smaller phyla include *Verrucomicrobia* (*Akkermansia*) and *Fusobacteria* (*Fusobacterium*) (Fig. 1) [24]. It has been shown that the proximal GIT is enriched in bacteria belonging to the phyla *Firmicutes* and *Proteobacteria*, in particular the genus *Lactobacilli*, compared to the distal GIT which mainly comprises of bacteria belonging to the phyla *Bacteroidetes* and *Firmicutes*, with particular attention to the *Akkermansia muciniphilia* species [25].

Intestine microbial composition is highly variable between individuals and is being continuously modified by both endogenous and exogenous factors [26]. The host genome has a central role in determining the composition of gut microbiota but many geographic and environmental factors such as diet, illness, lifestyle, hygiene and medication can contribute to changes in the population [27–29]. Reports have shown dietary modification can account for 57% of the variations in gut microbiota compared to host genetic variations that may account for as little as 12% [30]. There is now a considerable amount of data to suggest that disruption of gut microbiota may give rise to many inflammatory diseases such as obesity, inflammatory bowel disease, T2DM, arthritis and cancer [31].

As mentioned previously, the gut microbiota are becoming increasingly recognized as having a fundamental role in human physiology and health, [32] with resulting direct or indirect host effects [33]. Mechanisms include:- the fermentation of non-digestible substrates like dietary fibre and endogenous intestinal mucus to produce short chain fatty acids (SCFAs); the modulation of the immune and inflammatory response; regulation of neuronal signaling; regulation of integrity and mobility of the gut barrier; biosynthesis of vitamins, steroid hormones, and neurotransmitters; metabolism of branched chain amino acids (BCAAs), bile salts, and drugs; and the regulation of the hepatic production of triglycerides by suppressing lipoprotein lipase inhibitors [34–37].

## Microbiome influence on obesity and T2DM

Diversity is key to a healthy gut allowing for microbe redundancy with multiple microbes capable of performing similar functions. Disruption in specific host microbial populations may be more important than overall phylogenetic ratios, resulting in changes in the production of SCFAs and metabolites that directly influence glucose and insulin regulation [38–40]. Suggested mechanisms for this development which will be discussed throughout this review and include immune dysregulation; altered energy regulation; altered gut hormone regulation; and pro-inflammatory mechanisms [41]. Body weight is not controlled by the calories that are ingested but rather by the calories that are absorbed [42]. When in calorie excess, adipose tissue cannot maintain its buffering capacity to store excess energy in the form of triglycerides, resulting in an overflow of lipids into the systemic circulation [43]. Increased lipid availability to non-adipose tissues such as the liver, skeletal muscle and pancreas contribute to ectopic fat storage and the development of insulin resistance. Secondly, adipose tissue generates inflammation triggering an increase in the production and secretion of pro-inflammatory cytokines and adipokines such as tumour necrosis factor.

(TNF), interleukins (IL-6) and monocyte chemoattractant proteins (MCP-1) which may also accelerate the development of peripheral insulin resistance and altered glucose homeostasis [43].

## Gut microbiota metabolites: the importance of SCFAs

SCFAs are small organic monocarboxylic acids and are the major microbial metabolites produced during anaerobic carbohydrate fermentation in the intestine by acting as vital components in microbe-to-host signaling pathways [44]. Acetate, butyrate and propionate constitute greater than 95% of the total SCFA content [35, 45]. By-products of carbohydrate fermentation include the colonic gases (hydrogen (H_2_), carbon dioxide (CO_2_) and methane (CH_4_)) which can have inhibitory effects, limiting SCFA production [35, 45, 46]. The species *Methanobrevibacter Smithii* (a H_2_-using methanogen) prevents the accumulation of H_2_ by combining together H_2_ and CO_2_, producing CH_4_, allowing for the continued carbohydrate fermentation resulting in greater SCFA production and the availability of calories to the host [47].

**Fig. 2:**
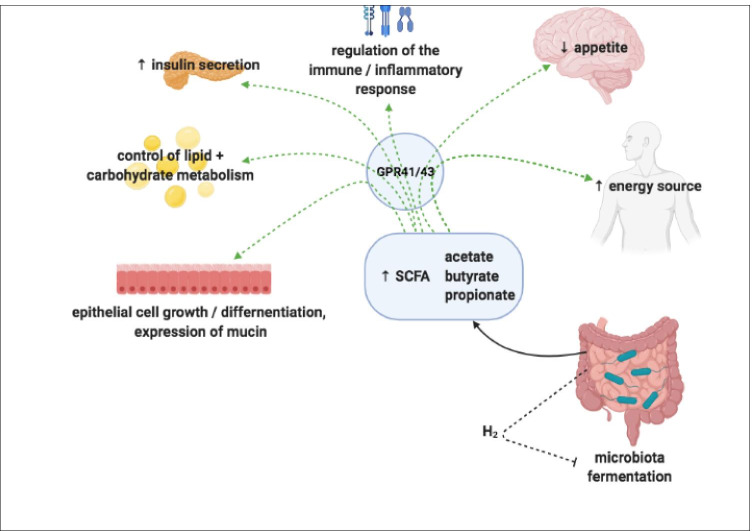
Overview of SCFAs in host metabolism. SCFAs provide an important energy source; facilitate hepatic control of lipids and carbohydrates; aid the transportation and metabolism of epithelial cells; influence epithelial cell growth and differentiation; promote the expression of mucin to strengthen the intestinal barrier; stimulate host inflammatory pathways; and regulate the immune system for the elimination of pathogens

The roles of SCFAs in the host include constituting an important energy source providing as much as 10% of the daily energy requirement [48, 49]; facilitate hepatic control of lipids and carbohydrates; aid the transportation and metabolism of epithelial cells; positively influence epithelial cell growth and differentiation [50, 51]; promote the expression of mucin to strengthen the intestinal barrier [52]; have anti-inflammatory properties reducing the secretion of pro-inflammatory cytokines and chemokines [53]; and serve as immune stimulators to condition tissue and immune cells to better eliminate pathogens [54] (Fig. 2).

SCFAs are predominantly produced from gut microbiota such as the genera *Prevotella*, *Ruminococus, Coprococcus*, and *Roseburia,* and the species *Akkermansia muciniphilia* and *Eubacterium rectale* [55]. During host fasting periods, intestinal microbes have adapted such that *Akkermansia muciniphilia* can degrade intestinal mucus, increasing the local availability of N-Acetylglucosamine, N-acetylgalactosamine, fructose and galactose which can serve as essential substrates for continued microbial fermentation [45, 56].

## SCFAs in metabolic pathways

**Fig. 3:**
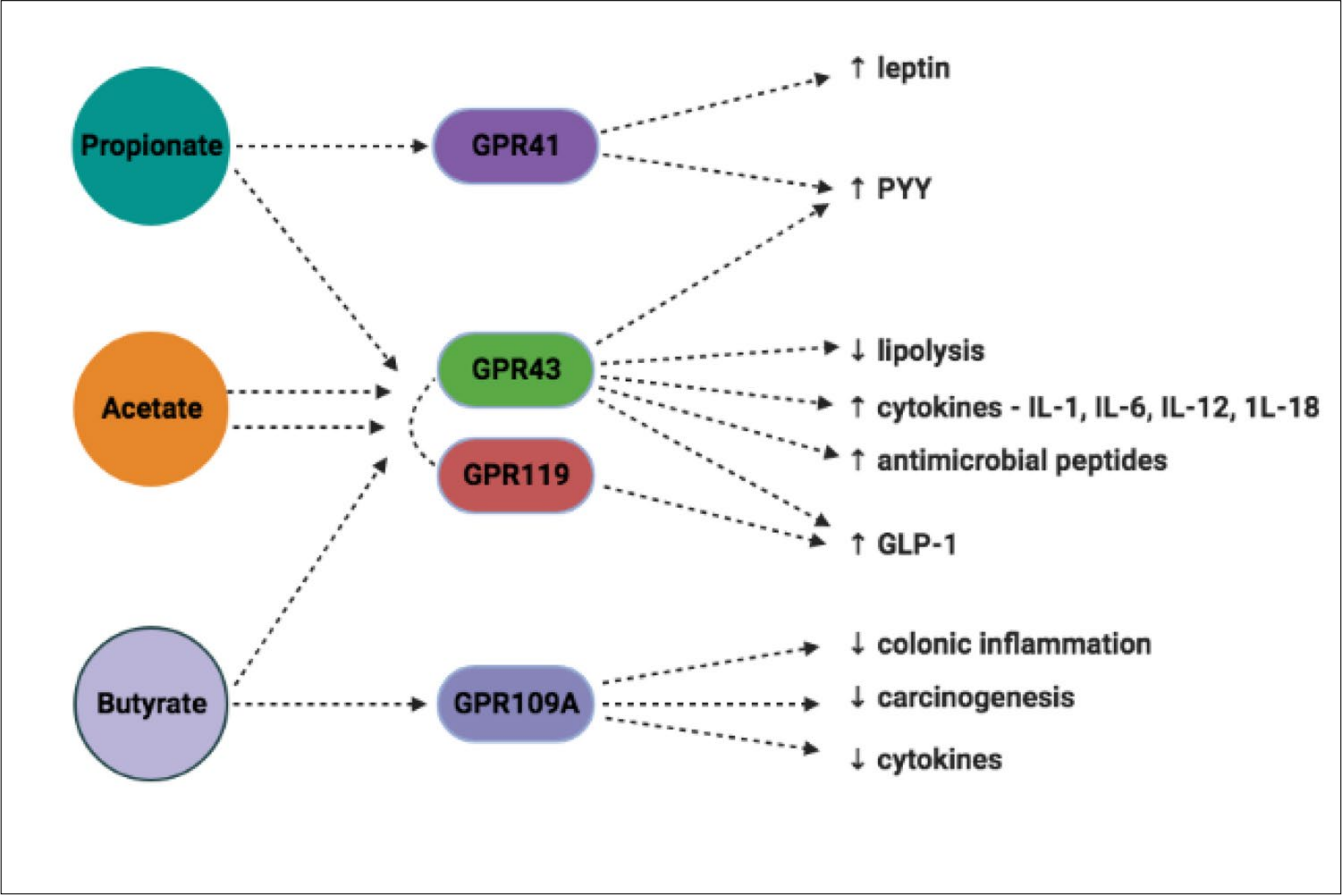
Main SCFAs and their roles in host signaling pathways. SCFAs perform a central role in metabolic pathways linking with selected GPRs. GPRs are abundant in adipocytes, intestinal immune cells, gut epithelial cells and pancreatic β-cells. Propionate primarily activates GPR41 and butyrate activates GPR109A. GPR43 and GPR119 can be activated by acetate, butyrate and propionate at similar rates

SCFAs perform a central role in metabolic pathways acting as signaling molecules by linking with selected G-protein-coupled receptors (GPRs): GPR41, GPR43 (also termed FFAR3 and FFAR2) GPR119 and GPR109A which are abundant in adipocytes, intestinal immune cells, gut epithelial cells and pancreatic β-cells [57–61]. Propionate primarily activates GPR41, butyrate activates GPR109A however GPR43 and GPR119 can be activated by acetate, butyrate and propionate at similar rates [61, 62] (Fig. 3).

Activation of GPR41 and GPR43 induces the secretion of peptide tyrosine-tyrosine (PYY), a short peptide that is released into the ileum and colon to reduce appetite by directly affecting the central nervous system. GPR41 coupling can stimulate adipocytes to express leptin [63, 64], resulting in the inhibition of the release of neuropeptide Y (NPY), a hypothalamic neurotransmitter that stimulates eating. This was first demonstrated using GPR41 deficient mice that display significantly lower leptin levels than corresponding wild-type mice [65].

GPR43 activation leads to positive homeostatic effects on several metabolic pathways:- the release of glucagon-like peptide 1 (GLP-1) from entero-endocrine L-cells which stimulate glucose-medicated insulin release from pancreatic β-cells, suppress pancreatic α-cell glucagon secretion, protect β-cells from apoptosis, promote β-cell proliferation and increase GIT transit time [66–68]; acetate activation directly reduces lipolysis in adipocytes leading to decreased plasma-free fatty acid levels [69]; promotion of the production of the antimicrobial peptides RegIIIγ and β-defensin in intestinal epithelial cells; and the generation of immunity-related cytokines such as IL-1, IL-6, IL-12 and IL-18 [70, 71].

GPR43 is mainly expressed in immune tissue and white adipose tissue as demonstrated in wild-type mice. Activation of GPR43 in white adipocytes reduces insulin-induced protein kinase B (AKT)-activation consequently suppressing fat accumulation. GPR43-deficient mice are phenotypically obese in contrast to mice who overexpress GPR43, specifically in adipose tissue. This cohort maintain their lean status even when subjected to high calorific feeding. Germ-free (GF) and antibiotic treated mice both had normal phenotypes suggesting the importance of gut microbiota as deliverers of GPR activating SCFAs [72].

GPR119 agonists reduce circulating blood glucose levels by promoting the intestinal secretion of GLP-1, improving pancreatic β-cell function and insulin secretion [60]. GPR109A stimulation suppresses colonic inflammation and carcinogenesis, promoting anti-inflammatory aspects of colonic macrophages and dendritic cells inducing the differentiation of regulatory and IL-10 producing T-cells. GPR109A expression decreases in the absence of gut microbiota [73, 74].

## Acetate

The most abundant SCFA in the GIT is acetate which is readily absorbed, transported to the liver and acts as a substrate in cholesterol synthesis. Very little metabolism occurs within the colon. Acetate behaves as a substrate and signaling molecule in the processes of cholesterol synthesis, lipogenesis, host appetite and glucose homeostasis. *Methanobrevibacter smithii* can further exacerbate lipogenesis to the detriment of host adiposity, as it is heavily involved in the bacterial fermentation of fructans producing large amounts of acetate [75].

On entering the systemic circulation, the presence of acetyl-CoA synthetase in adipose tissue allows for the immediate usage as a substrate in lipogenesis [76]. Acetate may have the ability to influence host appetite by manipulating the expression profiles of regulatory neuropeptides situated in the hypothalamus through activation of the Krebs cycle [43, 77]. Using a rodent model, Perry et al. demonstrated that increased levels of acetate led to an elevated production of ghrelin, the stimulation of parasympathetic activity which thus increased food intake and the promotion of glucose-stimulated insulin secretion [78].

## Butyrate

Butyrate has many important properties in host homeostasis:—acts as the main substrate in colonocyte metabolism [76, 79]; regulates cell proliferation and differentiation [62, 79]; induces apoptosis of colonic cancer cells [59, 62, 80, 81]; activates intestinal gluconeogenesis (80); induces the inflammation cascade; provides protection against oxidative stress [81]; and maintains the permeability of the gut barrier [59, 62, 82].

Butyrate is oxidised in the mitochondria of colonocytes and contributes as a substrate in the Krebs cycle for adenosine triphosphate (ATP) production. Catalysing enzymes in this process are down-regulated in GF-mice, leaving significantly decreased levels of ATP in GF-colonocytes. This highlights the potential stimulating role for butyrate-producing microbiota [83–85].

Butyrate can affect DNA methylation, proliferation and differentiation in colonic epithelial cells by inhibiting histone deacetylase and suppressing nuclear factor kappa B (NF-kB) activation [86]. NF-kB is a transcription factor in control of gene expression encoding pro-inflammatory cytokines, chemokines, inducible inflammatory enzymes, adhesion molecules, growth factors and some acute phase proteins and immune receptors [59, 87].

Butyrate exerts anti-inflammatory effects through the inhibition of interferon-y production, signaling pathways and the up-regulation of peroxisome proliferator-activated receptor gamma (PPARγ). PPARγ is a ligand-activated transcription factor that is highly expressed in colonic epithelial cells and is thought to activate the anti-inflammatory cascade [59].

## Propionate

Propionate influences aspects of glucose homeostasis, inhibits hepatic cholesterol synthesis [62, 88], exerts anti-inflammatory effects by the promotion of regulatory T-cell differentiation and IL-10 production [53] and has the ability to reduce host appetite. It has been demonstrated to have competing and opposite effects on gluconeogenesis [89] acting as both a substrate and as an inhibitor [88]. The inhibiting effect may be related to its metabolic intermediaries, methymalonyl CoA and succinyl CoA, which are specific inhibitors of pyruvate carboxylase. Propionate may influence hepatic glucose metabolism indirectly by lowering plasma fatty acid concentration [90], which is closely related to the rate of gluconeogenesis [91].

Propionate stimulates the intestinal release of the satiety hormone PYY and GLP-1 coupling with GPRs [43]. De Vadder et al., demonstrated propionate supplementation can result in the reduction of weight, abdominal adipose tissue, hepatic fat and assist with insulin sensitivity maintenance [80]. Alhabeeb et al., also displayed the beneficial effects of propionate supplementation in healthy volunteers exhibiting increased satiety and reduced appetite, as measured by visual analogue scales [92].

## Other gut microbiota fermentation products

BCAAs, succinate, ammonia, amines, phenol and indole are other important gut microbiota metabolites which also behave as nutrients, messenger molecules and have the ability to shape host pathophysiology. They are mainly derived from protein metabolism, fermentation of aromatic amino acids and dietary fibre [93]. The most abundant amino acid fermenting bacteria belong to the genera *Clostridium*, *Bacteroides*, *Lactobacillus*, *Streptococcus*, *Propionibacterium* and *Fusobacterium* [94, 95].

Bacterial fermentation of dietary fibre produces large amounts of succinate, reported as an unsuspected bacterial metabolite with the ability to improve glycaemic control through the activation of intestinal gluconeogenesis [96]. Increased BCAA plasma levels correlate with specific bacterial species such as *Prevotella copri* and *Bacteroides vulgatus* and have been shown to be characteristic of individuals with insulin resistance [97]. Increases in a small number of essential amino acids including the BCAAs (leucine, valine, and isoleucine), and the aromatic amino acids (phenylalanine and tyrosine), have been reported to be associated with a five-fold increased risk of developing T2DM [98].

Indolepropionic acid, generated from bacterial aromatic amino acid catabolism, is strongly correlated with host dietary fibre intake and appears to reduce the risk of developing T2DM. It exerts potent anti-oxidative activity and has radical scavenging properties in vitro, suggesting it has the ability to provide protection for the pancreatic β-cell and possibly from amyloid accumulation [99]. Indolepropionic acid may also aid the modulation of incretin secretion from entero-endocrine L-cells by inhibiting voltage-gated potassium channels. This affects the action potential properties of L-cells resulting in enhanced calcium entry, triggering GLP-1 secretion. However, when stimulated over a longer period of time, it leads to the inhibition of mitochondrial metabolism creating a reduction in intracellular ATP concentration. This induces the opening of ATP-sensitive potassium channels, hyperpolarising the plasma membrane and slowing GLP-1 secretion [100, 101].

Lastly, imidazole propionate, produced from the degradation of histidine by gut microbiota impairs the ability of cells to correctly respond to insulin. Imidazole propionate inhibits the intracellular insulin receptor signalling cascade by activating the p38γ–p62– mammalian target of rapamycin complex 1 (mTORC1) pathway which inhibits the formation of the insulin receptor substrate protein and mTORC1 complex. mTORC1 is an integral part of the intracellular cascade and regulates various metabolic pathways, including the insulin receptor cascade [102].

## Bile acids and gut microbiota and the beneficial effects on the host

Bile acids (BAs) are steroid carboxylic acids produced in perivenous hepatocytes primarily from the hydroxylation of cholesterol which is controlled by cytochrome P450 enzyme cholesterol 7α hydroxylase (CYP7A1). Before being secreted for storage in the gallbladder, primary BAs are conjugated to glycine further enhancing their hydrophilicity. Entero-hepatic circulation enables 95% of BAs to be reabsorbed from the distal ileum, allowing time for the interaction of gut microbiota and primary BAs to produce secondary BAs [103]. BAs have multiple functions including the facilitation of the digestion and absorption of dietary fats and lipid-soluble vitamins in the small intestine; maintenance of the intestinal barrier; and controlling metabolic pathways by acting as signaling molecules for the regulation of triglyceride, cholesterol, glucose and energy homeostasis [104].

**Fig. 4:**
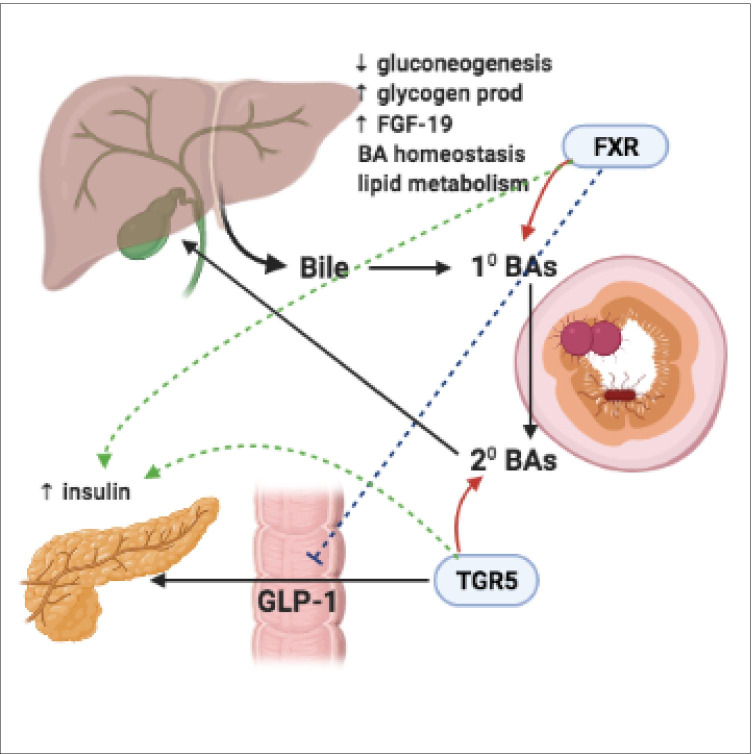
BA synthesis and signalling pathway. BAs are produced in perivenous hepatocytes primarily from the hydroxylation of cholesterol. Before being secreted for storage in the gallbladder, primary BAs are conjugated to glycine further enhancing their hydrophilicity. Entero-hepatic circulation enables 95% of BAs to be reabsorbed from the distal ileum, allowing time for the interaction of gut microbiota and primary BAs. Gut microbiota convert host-derived primary BAs into secondary BAs. Primary BAs bind to the FXR receptor, secondary BAs bind to the TGR5 receptor. FXR signaling can decrease gluconeogenesis, promote glycogen production and enhance the secretion of gut-derived hormones such as FGF-19. FGF-19 has the ability to regulate BA synthesis, lipid and glucose metabolism. TGR5 receptor activation results in GLP-1 secretion from intestinal L-cells. FXR signaling inhibits GLP-1. BA-TGR5 and BA-FXR receptor signaling stimulates insulin production from pancreatic β-cells

Gut microbiota play a key role in BA synthesis, modification and signaling by converting host-derived primary BAs into secondary BAs, and by deconjugation through the enzymatic activity of bile salt hydrolases [105]. The predominant microbiota that contribute in BA pathways are from the genera *Lactobacillus*, *Bifidobacteria*, *Enterobacter*, *Bacteroides* and *Clostridium*. Primary BAs bind to the nuclear hormone farnesoid X receptor (FXR) whereas secondary BAs bind to G protein-coupled BA receptor 1 (TGR5) [103, 106]. BAs, acting through FXR signaling can decrease gluconeogenesis and promote glycogen production in the liver. FXR stimulation results in the secretion of gut-derived hormones, such as fibroblast growth factor 19 (FGF-19) which in turn regulates BA synthesis as well as lipid and glucose metabolism [103, 107, 108]. FGF-19 induces the synthesis of glycogen and inhibits glucose production [109]. TGR5 receptor activation results in GLP-1 secretion from intestinal L-cells, whereas FXR signalling inhibits GLP-1. Both BA-TGR5 and BA-FXR signalling stimulates insulin production from β-cells in the pancreas. Glucose-stimulated insulin release is additionally promoted by BA-TGR5 signalling in α-cells, which promotes the conversion of pro-glucagon to GLP-1 and GLP-1 release (Fig. 4).

Gut microbiota can control BA synthesis by metabolising naturally occurring FXR antagonist tauro-β-muricholic acid resulting in the development of obesity, steatosis and impaired tolerance to glucose and insulin [110, 111]. Increased BA synthesis contributes to greater energy expenditure by the stimulation of brown adipose tissue and skeletal muscle via TGR5, and by increasing thyroid hormone production by activating type 2 deiodinase [106, 112]. In the hypothalamus, BA-TGR5 signalling mediates satiety. BA-TGR5 activation in immune cells results in the inhibition of the nucleotide-binding domain-like receptor protein 3 (NLRP3)-inflammasome (a multimeric protein complex) and attenuated inflammation [106].

## Disruption of the intestinal mucosal barrier by microbiota

The mucosal lining of the GIT acts as a natural barrier preventing undesirable interactions between the colonic epithelium, viruses, toxins and pathogenic bacteria [113]. Disruption of the GIT wall integrity allows for the translocation of toxins into the systemic circulation leading to metabolic endotoxaemia and results in low-grade inflammation, autoimmunity and oxidative stress, which have the potential for β-cell destruction and insulin resistance [38, 114]. Gut microbiota produce numerous organic compounds like nitrous oxide (NO), CH_4_, CO_2_, indole and hydrogen sulphide which possess pro and anti-inflammatory properties with the capability to alter GIT permeability [115].

**Fig. 5:**
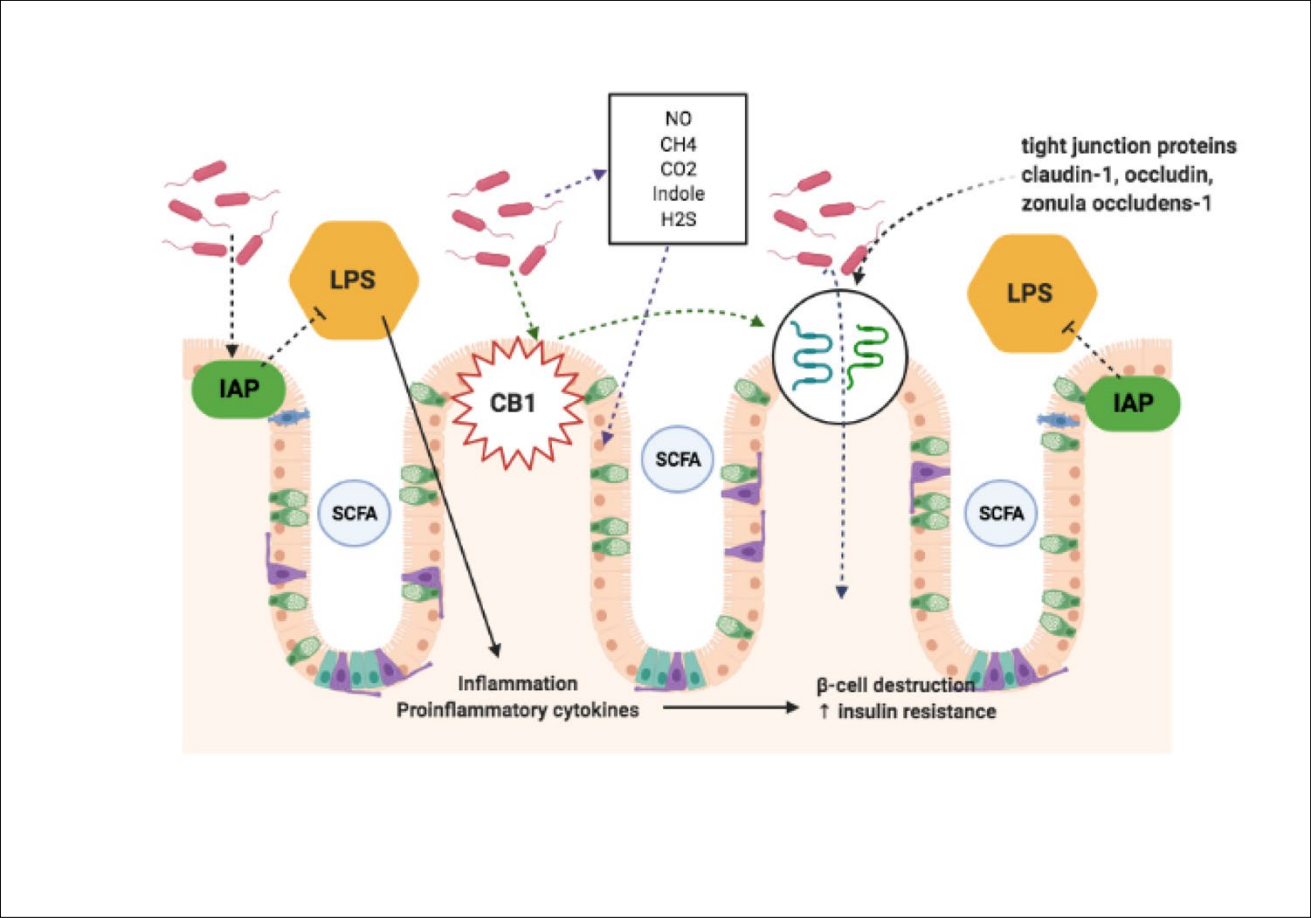
Gut microbiota interactions with the intestinal mucosal barrier. The mucosal lining of the GIT acts as a natural barrier preventing undesirable interactions. Disruption of the GIT wall integrity allows for the translocation of toxins into the systemic circulation leading to metabolic endotoxaemia resulting in low-grade inflammation, autoimmunity and oxidative stress. Gut microbiota produce numerous organic compounds like NO, CH_4_, CO_2_, indole and hydrogen sulphide with the capability to disrupt GIT permeability. GIT barrier function is maintained using:—appropriate localisation and distribution of tight junction proteins; the presence of a thick mucus layer covering the epithelial cells; up-regulation of the secretion of mucus from goblet cells; the presence of mucin-associated bacteria; a normal endocannabinoid system tone; and LPS detoxification by intestinal alkaline phosphatase

**Fig. 6:**
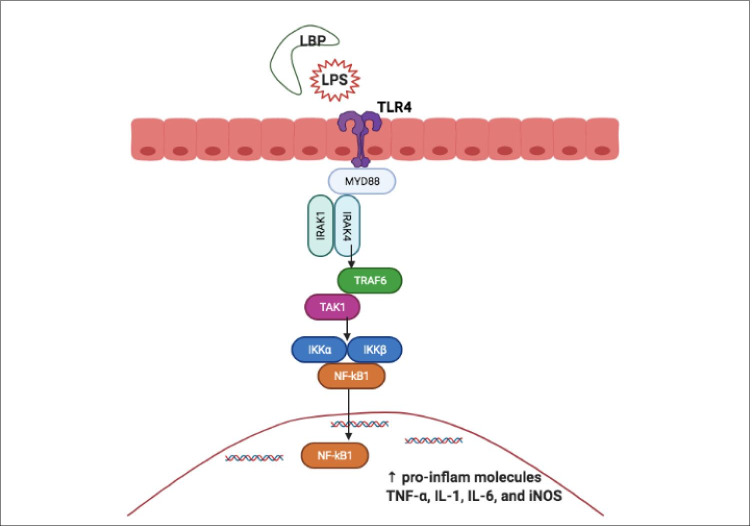
LPS triggering the inflammatory pathway. LPS is recognised and bound by LBP. Bacterial fragments and LPS are recognised by TLRs. LPS binds and activates TLR4, triggering dimerisation, and the recruitment of downstream adaptor molecules such as MyD88/MAL to mount an inflammatory response. Activated MyD88/MAL recruits IRAK), TRAF6, TAK1, JNK and IKK complexes. The IKK complex converges at NF-kB, which is maintained in the inactive state by IKKB. This, in turn, is degraded by proteasomes, resulting in the translocation of NF-kB into the nucleus, activating the release of pro-inflammatory molecules TNF-α, IL-1β, IL-6, and iNOS

GIT barrier function is maintained via several mechanisms:—appropriate localisation and distribution of tight junction proteins (claudin-1, zonula occludens-1 and occludin); the presence of a thick mucus layer covering the epithelial cells; up-regulation of the secretion of mucus from goblet cells by butyrate; the presence of mucin-associated bacteria [116, 117]; a normal endocannabinoid system tone; and lipopolysaccharide (LPS) detoxification by intestinal alkaline phosphatase (Fig. 5 and 6). The presence of SCFAs enhance gut barrier integrity [114, 118, 119]. Gut microbiota have the ability to disrupt intestinal tight junction proteins and alter alkaline phosphatase activity resulting in increased gut permeability. Gut microbes selectively act to modulate colonic expression of endocannabinoid receptor type-1 (CB1), which strongly influences gut permeability through effects on zonula occludens-1 and occludin [120, 121].

Obesity and T2DM are both conditions characterised by GIT barrier disruption leading to a significant increase in permeability, which correlates, with greater levels of LPS in the blood [122]. Brun et al., analysed cross-sectional samples of intestine obtained from obese mice that clearly demonstrated a decrease in the tight junction protein occludin and an irregular distribution of zonula occludens-1 [123]. Several species have been shown to benefit gut barrier function. *Faecalibacterium prausnitzii* and *Roseburia intestinalis* are important butyrate-producing microbes and are believed to protect against bacterial translocation either directly or through their ability to produce butyrate [124]. Increased levels of the genera *Bifidobacterium* have also been associated with reduced gut leakiness, allowing less LPS to translocate into the serum [122]. GIT motility and gut microbiota are closely interrelated and can significantly affect one another. Transplanting human gut microbes into GF-mice significantly shortened GIT transit time when subjected to a polysaccharide-rich diet [125]. SCFAs modulate colonic motility by stimulating the secretion of serotonin from gut enterochromaffin cells, in part through activation of the vagus nerve via the serotonin (5-HT_3_) receptor [126].

## Gut microbiota influence on the inflammatory response

Obesity and T2DM are characterised by chronic low-grade inflammation with abnormal expression and production of multiple inflammatory mediators such as increased levels of TNF, C-reactive protein (CRP), plasminogen activator inhibitor-1 and interleukins (IL-1, IL-6) [127–129]. The concept of inflammation in metabolic conditions was first proposed by Hotamisligil et al. who demonstrated that adipocytes can express the cytokine TNF-α and that this expression is particularly increased within obese animals. Neutralisation of TNF-α in these animals led to a decrease in insulin resistance. This is believed to be the first experiment to potentially expose a relationship between the expression and plasma concentration of a pro-inflammatory cytokine and insulin resistance [130].

Gut microbiota, acting through LPS activity can influence inflammation and insulin resistance. LPS is an essential component of the cell walls of Gram-negative bacteria such as the phylum *Bacteroidetes* [131–133]. The lipid A portion of LPS contains the relevant endotoxin activity and has differing levels of pro-inflammatory activity owing to the variability in the detailed lipid A structure. LPS from members of the families *Enterobacteriaceae* and *Desulfovibrionaceae* (phylum: *Proteobacteria*), exhibit an endotoxin activity that is 1,000-fold that of LPS from the family *Bacteroideaceae* (phylum: *Bacteroidetes* of which members of this phylum are the most numerous LPS producers in the gut) [134].

Dietary fat is transported from the intestine after being incorporated as triglycerides into chylomicrons which have a high affinity for LPS. Thus, the formation of chylomicrons aids the movement of LPS from intestinal cells into the circulation [131].

‘Metabolic endotoxemia’ is a condition characterised by a two to three-fold increase in circulating LPS levels [135]. After entering the circulation, LPS is recognised and bound by lipopolysaccharide binding protein (LBP), an acute-phase protein synthesised in the liver [136]. Bacterial fragments and LPS are recognised by toll-like receptors (TLRs) that are a family of key pattern recognition receptors that aid cells in the recognition of ligands such as endotoxin [132, 137, 138]. LPS binds and activates TLR4, triggering dimerisation, and the recruitment of downstream adaptor molecules such as myeloid differentiation primary response 88 adaptor-like (MyD88/MAL) to mount an inflammatory response [26] (Fig. 6).

Activated MyD88/MAL recruits the interleukin-1 receptor-associated kinase (IRAK), tumour necrosis factor receptor-associated factor 6 (TRAF6), transforming growth factor B-activated kinase 1 (TAK1), jun N-terminal kinase (JNK) and inhibitor of nuclear factor-kB kinase (IKK) complexes. The IKK complex converges at NF-kB, which is maintained in the inactive state by IkB. This, in turn, is degraded by proteasomes, resulting in the translocation of NF-kB into the nucleus, activating the release of pro-inflammatory molecules TNF-α, IL-1, IL-6, and inducible nitric oxide synthase (iNOS). The activation of serum kinases (JNK and IKK) can induce insulin receptor substrate (IRS-1) serine phosphorylation, resulting in insulin resistance [27].

TNF-α expression upregulates the transcription of suppressor of cytokine signaling 3 (SOCS-3) which binds to tyrosine 960 of the insulin receptor, preventing IRS-1 binding to the insulin receptor. IRS-1 is subsequently degraded leading to the disruption of the insulin signaling pathway and glucose transport (via GLUT-4) [136, 139]. The importance of the TLR-4 pathways in worsening metabolic disease was confirmed by inducing a deletion of TLR-4 which subsequently prevented high fat diet (HFD) induced insulin resistance [140, 141].

LPS can also activate a local immune response via high-affinity binding to the NLRP3 inflammasome and NLRs expressed at high levels on the surface of macrophages and dendritic cells [142]. They are believed to play a role in the development of leptin resistance [143], resulting in hyperphagia and weight gain further increasing fat intake, raising LPS and ongoing inflammation [144].

HFD feeding significantly alters the gut microbial composition by reducing the numbers of the genera *Bifidobacterium*, which have many physiologically positive effects [145]. Mice who consume a HFD supplemented with oligo-fructose have restored quantities of *Bifidobacterium* with associated decreased endotoxemia suggesting *Bifidobacterium* may improve intestinal permeability and lower circulating levels of endotoxin. The increase in *Bifidobacterium* correlates with improved glucose tolerance, glucose-induced insulin secretion, lower body weight and decreased production of inflammatory mediators [133, 146].

Obese rodents have two to three times greater levels of plasma LPS than non-obese counterparts and display low-grade systemic inflammation. Injection of the species *Escherichia coli* LPS subcutaneously into wild-type mice fed on normal chow led to the development of inflammation, obesity, fasted glycaemia and insulinaemia. Importantly, in cluster of differentiation 14 (CD14)-knockout mice, in whom LPS cannot be recognised, there was a delay or even a complete lack of development of most features of metabolic disease induced by a HFD or LPS infusion [135].

## Antibiotic induced disruption of the microbiota

Antibiotics can strongly influence the composition of the gut microbiota for up to two years after administration [147]. Disruption caused by antibiotic treatment can induce a stress response which facilitates the transfer of drug resistant genes to virulent species leading to drug resistance [148]. A recently performed meta-analysis concluded an increased risk of childhood obesity in those children exposed to more than one antibiotic treatment within their first six months of life [149]. A population-wide case–control study in Denmark demonstrated a positive relationship between antibiotic exposure and the development of T2DM years later and a relationship between a T2DM diagnosis and the number of antibiotic prescriptions. Antibiotics may predispose patients to T2DM, however the authors suggest caution because T2DM patients are possibly more vulnerable to developing infections in the years prior to diagnosis [150].

Vrieze et al., analysed the effects of antibiotic treatment on gut microbiota and its effect on metabolic parameters in patients diagnosed with obesity and insulin resistance. Vancomycin significantly reduced microbial diversity, with particular decreases in the abundance of *Firmicutes*, mainly butyrate-producers, with corresponding increases in the phylum *Proteobacteria*, specifically the genera *Lactobacillus*. These microbial changes were accompanied by an overall decrease in peripheral insulin sensitivity. No effect was observed with amoxicillin [151].

## Impact of the gut microbiota on the gut-brain axis

The gut-brain axis is a bi-directional signalling pathway regulating metabolism through balancing food intake and energy expenditure [152] whilst also influencing behaviour and brain function [153]. The gut signals to the brain via the central nervous system or acting through microbiota-derived metabolites, which have a role in controlling appetite directly or indirectly. Metabolites such as GLP-1, PYY, leptin and ghrelin modify gut hormone secretion, which can impact hypothalamic neuroendocrine pathways [78, 154, 155]. In particular, GLP-1 and PYY have receptors expressed in the brain involved in the regulation of host energy balance [155].

**Fig. 7:**
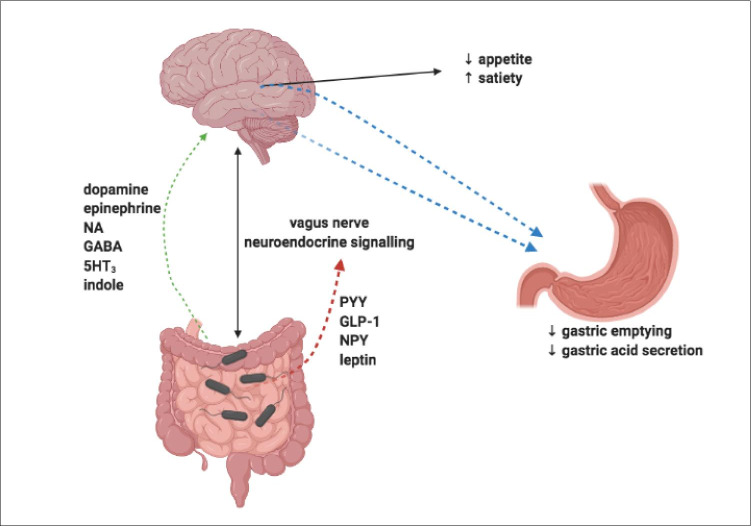
Gut microbiota effects on the gut-brain axis. The gut-brain axis is a bi-directional signalling pathway influencing food intake, energy expenditure, behaviour and brain function. The gut signals to the brain via a combination of the central nervous system and microbiota-derived metabolites. PYY modulates appetite and satiety by the suppression of NPY and activating POMC neurons in the ARC or by delaying gastric emptying. GLP-1 also regulates appetite via effects on POMC and NPY neurons in the ARC and contributes to the inhibition of gastric emptying and gastric acid secretion. Dopamine, epinephrine, norepinephrine, gamma-aminobutyric acid, serotonin and indole are derived by gut microbiota and may affect a persons’ dietary preference

PYY modulates appetite and satiety by the suppression of NPY and activating proopiomelanocortin (POMC) neurons in the arcuate nucleus (ARC) or by delaying gastric emptying [156, 157]. GLP-1 also regulates appetite via effects on POMC and NPY neurons in the ARC and contributes to the inhibition of gastric emptying and gastric acid secretion [158, 159] (Fig. 7).

There is growing evidence that dopamine, epinephrine, norepinephrine, gamma-aminobutyric acid, serotonin, indole metabolites and other neurotransmitters derived by gut microbiota could affect a persons’ dietary preference [153].

## Future Direction

The discovery of a link between gut microbiota and global metabolic disorders such as obesity and T2DM is undeniably exciting with the tantalizing possibility of targeting therapeutics for the production of personalised treatments. Well-designed, tightly controlled prospective human and animal studies are still required to establish causality between microbiota and metabolic disease. The vast majority of current gut microbiota human studies represent simple associations only; from which it is extremely difficult to interpret any firm causal conclusions. However, by using next-generation technology such as metagenomics and metabolomics to target microbial combinations with similar functions, we should develop a better understanding of the relationship between gut microbiota and metabolic disease.

Interventional studies surrounding microbiota transfer (faecal microbiota transplant, FMT) for metabolic disorders remain in their infancy, by using mainly GF-rodent models. These models provide crucial mechanistic information, however, it remains difficult to directly translate this data to humans with their inherent heterogeneity. We have already seen that gut microbiota can be disrupted by both host and environmental factors, so further investigations are required to fully appreciate this relationship.

Furthermore, by fully understanding the interplay between gut microbiota and metabolic disease, and by using methods such as microbiota transfer, pre/probiotic supplementation and dietary manipulation, we can design well-defined prospective studies to develop personalised treatments.

## Conclusion

The increasing worldwide epidemic of obesity and T2DM is a cause of significant morbidity and cost. Both conditions are complex metabolic disorders with varying aetiology and early scientific evidence suggests that gut microbiota play a pivotal role in their development. The advancement of microbial analysis techniques and the combined usage of rodent and human models has allowed the ongoing investigation of the gut microbiota, as part of the patho-aetiology of T2DM and obesity, to become more achievable.

Gut microbiota acting both directly and indirectly through the degradation products of intestinal fermentation have the ability to manipulate host homeostasis, metabolic and inflammatory pathways. This influence can be both beneficial and detrimental to the host. This review has provided valuable insight into the many complex interactions that the gut microbiota initiate. The review appreciates the importance of the SCFAs, more specifically, acetate, butyrate and propionate, as well as bile acids, in initiating the inflammatory cascade, the disruption caused to microbiota associated with antibiotic treatments, their ability to control the permeability of the mucosal barrier and subsequent manipulation of the gut-brain axis to alter host behaviour.

Further detailed scientific work is necessary towards establishing the gut microbiota’s role in its ability to control the disease processes for the future purpose of treating both obesity and T2DM.

## Data Availability

Data sharing not applicable to this article as no datasets were generated or analysed during the completion of this review.

## Abbreviations

AKT: Protein kinase B
ARC: Arcuate nucleus
ATP: Adenosine triphosphate
BA: Bile acid
BCAA: Branched chain amino acid
BMI: Body mass index
CB1: Cannabinoid receptor type-1
CD14: Cluster of differentiation 14
CH_4_: Methane
CO_2_: Carbon dioxide
CoA: Coenzyme A
CRP: C-reactive protein
CYP7A1: Cytochrome P450 enzyme cholesterol 7α-hydroxylase
DNA: Deoxyribonucleic acid
FFAR: Free fatty acid receptor
FGF-19: Fibroblast growth factor 19
FXR: Farnesoid X receptor
GF: Germ free
GIT: Gastrointestinal tract
GLUT-4: Glucose transporter type-4
GLP-1: Glucagon-like peptide 1
GPR: G protein coupled receptor
H_2_: Hydrogen
HFD: High fat diet
5-HT_3_: Serotonin
IKK: Inhibitor of nuclear factor-kB kinase
IL: Interleukin
iNOS: Inducible nitric oxide synthase
IRAK: Interleukin-1 receptor-associated kinase
IRS-1: Insulin receptor substrate 1
JNK: Jun N-terminal Kinase
Kg: Kilograms
LBP: Lipopolysaccharide binding protein
LPS: Lipopolysaccharide
MCP-1: Monocyte chemoattractant protein-1
mTORC1: Mammalian target of rapamycin complex 1
MyD88: Myeloid differentiation primary response 88
MyD88 MAL: Myeloid differentiation primary response 88 adaptor-like
NF-kB: Nuclear factor kappa B
NLRP3: Nucleotide-binding domain-like receptor protein 3
NLRs: Nucleotide-binding oligomerization domain-like receptors
NO: Nitrous oxide
NPY: Neuropeptide Y
POMC: Proopiomelanocortin
PPARy: Peroxisome proliferator-activated receptor gamma
PYY: Peptide tyrosine-tyrosine
RNA: Ribonucleic acid
SCFA: Short chain fatty acid
SOCS-3: Suppressor of cytokine signalling 3
TAK1: Transforming growth factor B-activated kinase 1
TGR5: G protein-coupled bile acid receptor 1
TLRs: Toll-like receptors
T2DM: Type two diabetes mellitus
TNF: Tumour necrosis factor
TRAF6: Tumour necrosis factor receptor-associated factor 6
UN: United Nations
WHO: World Health Organization
